# NOREC4DNA: using near-optimal rateless erasure codes for DNA storage

**DOI:** 10.1186/s12859-021-04318-x

**Published:** 2021-08-17

**Authors:** Peter Michael Schwarz, Bernd Freisleben

**Affiliations:** Department of Mathematics and Computer Science, Philipps-Universität Marburg, 35032 Marburg, Germany

**Keywords:** DNA storage, Erasure code, Near-optimal rateless erasure code, Raptor code, Online code, LT code

## Abstract

**Background:**

DNA is a promising storage medium for high-density long-term digital data storage. Since DNA synthesis and sequencing are still relatively expensive tasks, the coding methods used to store digital data in DNA should correct errors and avoid unstable or error-prone DNA sequences. Near-optimal rateless erasure codes, also called fountain codes, are particularly interesting codes to realize high-capacity and low-error DNA storage systems, as shown by Erlich and Zielinski in their approach based on the Luby transform (LT) code. Since LT is the most basic fountain code, there is a large untapped potential for improvement in using near-optimal erasure codes for DNA storage.

**Results:**

We present *NOREC4DNA*, a software framework to use, test, compare, and improve near-optimal rateless erasure codes (NORECs) for DNA storage systems. These codes can effectively be used to store digital information in DNA and cope with the restrictions of the DNA medium. Additionally, they can adapt to possible variable lengths of DNA strands and have nearly zero overhead. We describe the design and implementation of *NOREC4DNA*. Furthermore, we present experimental results demonstrating that *NOREC4DNA* can flexibly be used to evaluate the use of NORECs in DNA storage systems. In particular, we show that NORECs that apparently have not yet been used for DNA storage, such as Raptor and Online codes, can achieve significant improvements over LT codes that were used in previous work. *NOREC4DNA* is available on https://github.com/umr-ds/NOREC4DNA.

**Conclusion:**

*NOREC4DNA* is a flexible and extensible software framework for using, evaluating, and comparing NORECs for DNA storage systems.

## Background

Due to its very high storage density of theoretically 455 exabytes per gram (using 2 bits per nucleotide) [1] and its extraordinary longevity, deoxyribonucleic acid (DNA) is a promising medium for long-term and high-density storage of important data. However, since DNA as a biological medium is susceptible to a variety of mutations and read/write errors, and the cost for synthesizing and sequencing DNA is still a decisive factor, it is essential to use adequate coding schemes to correct errors and avoid unstable or error-prone DNA sequences when digital data is stored in DNA.

Several coding schemes were proposed to correct read/write errors and avoid error-prone DNA sequences. For example, Church et al. [1] as well as Goldman et al. [2] used different overlapping strategies to map digital data into DNA strands and support error correction. In these coding schemes, the bits 0 and 1 are mapped to two DNA bases each and thus error-prone combinations like long repeats of one base (called homopolymers) are avoided. Heckel et al. [3] proposed an index-based coding scheme, mapping data annotated with the corresponding index, without further modification of these data packets. Furthermore, near-optimal rateless erasure code (NOREC), also called *fountain codes*, are particularly interesting coding methods for DNA storage. For example, Erlich and Zielinski used the Luby transform (LT) code to achieve high-capacity and low-error DNA storage [4]. In their work, they leveraged the special property of fountain codes to be able to generate theoretically infinitely many different packets for a given input to find packets that satisfy the restrictions defined for their DNA storage approach. Since LT is the most basic NOREC, there is a large untapped potential for improvement in using NORECs for DNA storage.

Ping et al. [5] developed Chamaeleo, a framework that provides multiple DNA storage codes. While Ping et al. focus on a variety of conventional (non-fountain) codes presented in the literature, they also include the LT implementation used by Erlich and Zielinski. In contrast to our work, Chamaeleo does not include means to change, adapt, and integrate modified or new error rules. Furthermore, the missing framework-wide error simulation does not permit an extensive comparison of the usability of the implemented methods for real DNA storage.

In this paper, we present *NOREC4DNA*, a software framework to use, test, compare, and improve coding schemes for DNA storage. While *NOREC4DNA* focuses on fountain codes, regular coding schemes can be compared as well. Besides multiple extensible restriction rules, we implemented the three most common fountain codes, namely LT [6], Online [7], and Raptor [8]. The contributions of this paper are as follows:

- We present a novel framework based on NORECs, called *NOREC4DNA*, to flexibly generate sequences for DNA storage that satisfy user-defined restrictions.
- *NOREC4DNA* allows detailed comparisons of various coding schemes for storing data in DNA.
- We show that NORECs belong to the most suitable codes for DNA storage; Raptor codes yield the best results across all tested metrics.

The paper is organized as follows. “Near-optimal rateless erasure codes” section gives an overview of the fountain codes and technologies used in *NOREC4DNA*. “Implementation” section presents the design and implementation of *NOREC4DNA*. We present experimental results generated using *NOREC4DNA* in “Results” section. Finally, “Conclusion” section concludes the paper and outlines areas of future work.

### Near-optimal rateless erasure codes

NORECs can be used to generate theoretically infinitely many coding symbols (in practice, some limitations apply). Furthermore, only a small number of $(1+\epsilon ) * n$ encoded symbols have to be correctly received to fully reconstruct the original information. Since it does not matter which (and in which order) symbols are received as long as a sufficient number of symbols is received - just like a bucket under a fountain that does not care about which drops of water it collects—these codes are also known as *fountain codes*.

Typically, fountain codes are applied as follows. First, a distribution function is used to determine a degree for each data packet. These packets are filled with random chunks using XOR according to their degree and then transmitted to the recipient over an erasure channel. After receiving a sufficient subset of the transmitted packets, the receiver computes the original data using the received packets and an indication of the chunks contained in them. To reconstruct the encoded data, the receiver either has to know the list of chunks mixed into a given packet (e.g., as part of the transmission) or has to know the distribution function as well as the seed used to initialize the random number generator used during encoding.

#### Luby transform codes

**Fig. 1:**
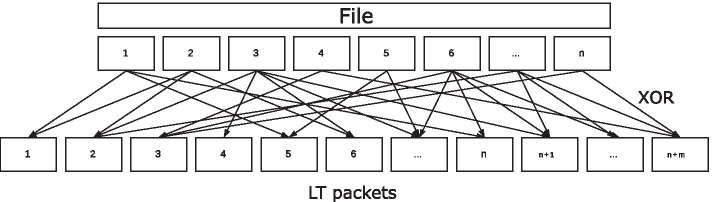
Encoding using LT

The LT code [6] proposed by Luby is considered to be the most fundamental and pioneering fountain code. LT divides the original file into *n* equally long ‘chunks’ that are then combined into packets using the XOR operator, as shown in Fig. 1.

The encoding process can be summarized as follows:

- Choose the degree *d* from the probability distribution function *p*(.).
- Select *d* evenly random distributed different chunks.
- Generate the resulting packet $t_i$ by combining all selected chunks using the XOR operator.
- Add reconstruction information (selected chunk number or seed) to the packet.

Luby presents two distribution functions for his LT coding schemes [6]. The first distribution function, called ‘IdealSoliton’ distribution, was proven by Luby to be mathematically ideal. It operates on integers from 1 to *N*, with *N* as the only parameter of the function. It is defined as follows:

1 $$\begin{aligned} \begin{aligned} \displaystyle \rho (1)&={\frac{1}{N}},\\ \displaystyle \rho (k)&=\frac{1}{k(k-1)}\qquad (k=2,3, \ldots ,N).\, \end{aligned} \end{aligned}$$

**Fig. 2:**
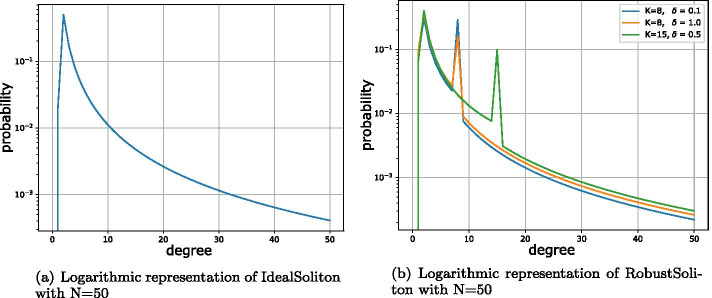
Soliton distributions

As shown in Fig. 2a, this function has a single mode and then flattens down to the specified value *N*. Since only the parameter *N* can be selected, the position of the mode $\rho (2) = 0.5$ as well as its value cannot be changed.

According to Luby, this function is quite fragile and thus not suitable for practical applications. Therefore, he proposed a robust form of this distribution function, called ‘RobustSoliton’ distribution, which uses a set of elements $\tau (i)$ to extend the IdealSoliton distribution (see Eq. (2)), adding a spike to the mode at degree 1. In addition to the parameter *N* already defined in the IdealSoliton distribution, two additional parameters *K* and $\delta$ are introduced. While *K* with $K < N$ defines the integer position of the additional peak, $\delta$ describes the expected (real-valued) error probability.

2 $$\begin{aligned} \begin{aligned} \displaystyle \tau (i)&={\frac{1}{iK}},\qquad \qquad (i=1,2, \ldots ,K-1),\,\\ \displaystyle \tau (i)&={\frac{\ln (R/\delta )}{K}},\qquad (i=K),\,\\ \displaystyle \tau (i)&=0,\qquad \qquad \quad (i=K+1, \ldots ,N).\,\\ \displaystyle \text {with }&R = N/K\,\\ \end{aligned} \end{aligned}$$

Finally, as shown in Eq. (3), the values for $\tau (i)$ are added to $\rho (i)$ and normalized afterwards.

3 $$\begin{aligned} \begin{aligned} \mu (i) = \dfrac{\rho (i)+\tau (i)}{\sum _{j=1}^{N}(\rho (j)+\tau (j))} \end{aligned} \end{aligned}$$

Figure 2b shows the influence of the individual parameters on the two distribution functions. While the choice of *N* determines the maximum degree, the parameter *K* determines the position of the additional peak. With increasing $\delta$, the probabilities shift towards degree 2. Therefore, from the second peak and from all other degrees $i > 2$, small values of probability decrease to the mode at degree 2.

**Fig. 3:**
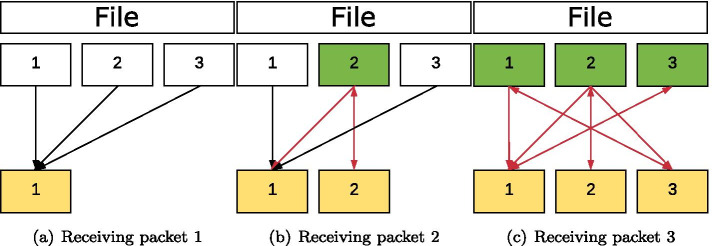
Decoding a file divided into three chunks

**Fig. 4:**
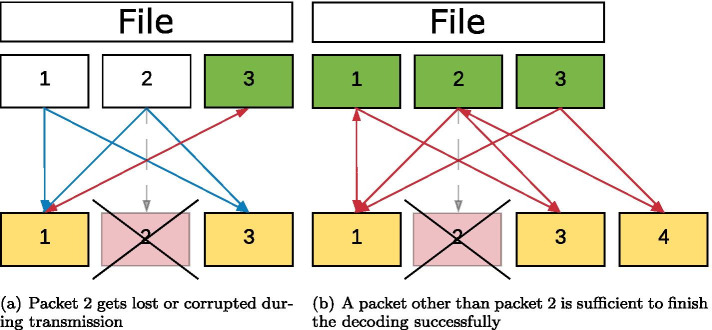
Decoding with packet loss

The decoding of the packets generated during the encoding can take place without prior sorting. For decoding, packets are first collected and combined (as far as possible) in such a way that the degree of these packets is reduced in each case. An illustration of decoding a file with three chunks is shown in Fig. 3. As shown in Fig. 4, the decoder can still reconstruct the information if packet 2 gets lost.

**Fig. 5:**
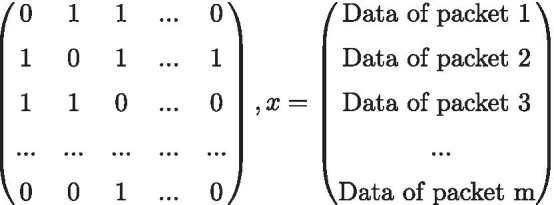
Decoding matrix after *m* received packets

Thanks to the special construction of fountain codes based on the XOR operator, it is possible to represent the decoding of all NOREC methods in the form of a linear equation system $Ax = b$, as shown in Fig. 5. This ensures that ambiguities during the decoding process, as shown in the previous example, can be avoided and thus the packets can be optimally reduced to the original message.

In *A*, all 1’s of a line *i* describe which chunks in the packet *i* were combined by the XOR operation. An example is shown in Eq. (4).

4 $$\begin{aligned} \begin{aligned} A[i]&= \begin{pmatrix} 0&1&0&0&1&1 \end{pmatrix}&\Leftrightarrow \\ b[i]&= {{{\veebar }}}_{j = 0}^{|A[i]|} A[i,j] \cdot Chunk[j] = Chunk[1] \veebar Chunk[4] \veebar Chunk[5] \end{aligned} \end{aligned}$$

#### Online codes

*Online Codes* proposed by Maymounkov [7] improve LT codes. Online codes address the main problem with LT codes, which is that LT codes cannot guarantee that after $(1+\epsilon )*n$ generated packets, all *n* parts of the original message were encoded in a sufficient manner. This problem is a manifestation of the coupon collector’s problem [9]. If a chunk of the original message has been encoded less frequently (e.g., only once or even zero times), these parts are much more vulnerable during decoding, since a loss of the packets containing these chunks cannot be compensated. Since fountain codes pseudo-randomly combine several chunks to packets, it can also happen that the lack of a single packet prevents numerous chunks from being reconstructed. To prevent this problem from occurring, Online codes follow a two-staged approach. First, auxiliary blocks are created in the so-called outer-encoding. Then, together with the message chunks, they are finally encoded into packets during the inner-encoding step. In particular, during the outer-encoding process, $M = \lceil 0.55 \cdot q \cdot \epsilon \cdot F\rceil$ auxiliary packets are created. Then, each chunk is randomly mixed into *q* different AUX packets using XOR [7]. Like the chunks of the file, the resulting *M* AUX packets are considered as regular inputs in the inner-encoding step. In this phase, a packet is generated by randomly selecting a degree *n* from the distribution function. As a result, selected from the union of the set of chunks and AUX blocks, *n* unique and equally distributed elements are mixed into this packet. This step is repeated infinitely or until a predefined condition occurs. Such a condition could be, e.g., reaching a certain number of created packets or successfully decoding with a decoder running simultaneously.

The process of decoding encoded content in Online codes is similar to the one described for LT codes. However, the decoding phases must be carried out in the reverse order of the encoding process.

**Fig. 6:**
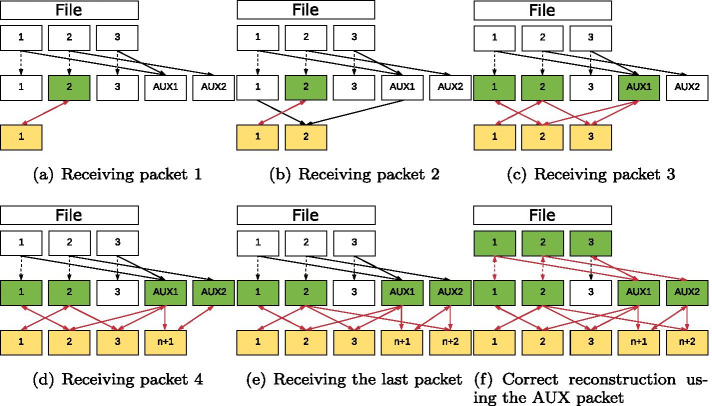
Online decoding of a file divided into three chunks with 5 generated packets and $q=1$

**Fig. 7:**
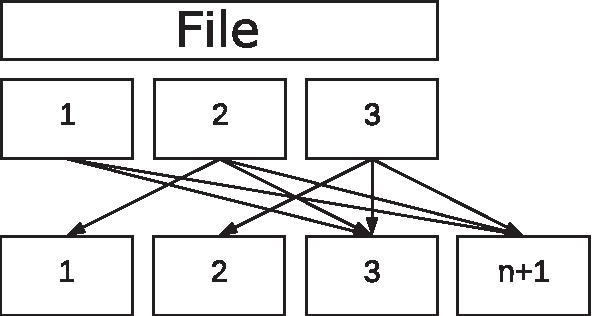
Reduction of the AUX packets

If the mapping of the chunks originally used per AUX packet are already available at the beginning of the decoding (e.g., after the first packet), the decoder can reduce the AUX packets and perform a mapping with the correct chunks during the actual transmission. A reduction of the example shown in Fig. 6 is illustrated in Fig. 7.

#### Raptor codes

*Raptor* codes developed by Shokrollahi [8] are the first fountain codes with (theoretically) linear encoding and decoding time. As the name Raptor codes (for rapid tornado) suggests, they are based on tornado codes, which can correct erasures with a fixed error rate. These codes require a constant number of blocks more than the Reed–Solomon codes [10], but are much faster in encoding and decoding. Meanwhile, there are several variants of the original method, e.g., R10 [11] or the RaptorQ code [12]. Depending on the procedure, purpose and area of use, various patents may apply [13, 14].

Similar to the previously mentioned Online codes, the Raptor encoding is based on a two-step process consisting of an outer- and an inner-encoding. While the inner-encoding (just like the inner-decoding) consists of an LT code, the outer-encoding of the Raptor code consists of one or a series of erasure codes with a fixed rate. A possible procedure of this outer-encoding phase is the encoding using a Gray sequence followed by an low density parity check (LDPC) code. Alternatively, a Hamming code or any other erasure code with a fixed rate can be used or combined. This approach combines the advantages of fixed rate coding with codes of the NOREC class.

In contrast to the fountain codes described so far, the Raptor encoding has a (theoretical) fixed upper limit of the number of possible chunks. This upper limit also limits the maximum degree of a packet. For this reason, Raptor codes use a fixed (parameter free) distribution function shown in Eq. (5). This function uses a random number of the given range $[1, 2^{20} = 1048576]$ to determine the degree of each packet.

5 $$\begin{aligned} \begin{aligned} f(x)= {\left\{ \begin{array}{ll} 1 &{} \text {for } x< 10241\\ 2 &{} \text {for } x< 491582\\ 3 &{} \text {for } x< 712794\\ 4 &{} \text {for } x< 831695\\ 10 &{} \text {for } x< 948446\\ 11 &{} \text {for } x < 1032189\\ 40 &{} \text {else} \end{array}\right. } \end{aligned} \end{aligned}$$

Data is encoded in two steps. First, the original data is used to create additional information for reconstruction using codes with a fixed rate. Second, the information generated is encoded into many packets using the LT coding technique. Although in practice various erasure codes exist for the first step, the Gray code combined with a subsequent LDPC code is mainly encountered. This can be explained by the simplicity of the Gray code and the properties of LDPC. With this combination (and especially since LDPC codes operate at the Shannon capacity), it is possible to successfully reconstruct a message with a very small overhead of packets (for 1000 chunks, approximately 1-2 additional packets are created using the non systematic approach) with a nearly 100% chance. The number of intermediate blocks to be generated in the first step of the Raptor code is calculated by the formulas shown in Eq. (6) and depends on the number of chunks.

6 $$\begin{aligned} \begin{aligned} f(k) =&x \in {\mathbb {N}} \text { with } x \cdot (x-1) \ge 2 \cdot k \text { and }\\&\exists ! j< x \text { with } j \cdot (j-1) \ge 2k\\ g(k) =&x \in {\mathbb {N}} \text { with } x \text { is prime and }\\&x \ge \lceil 0.01 \cdot k + f(k) \rceil \text { and }\\&\exists ! j \text { with } j \text { is prime and }\\&j< x \text { and } j \ge \lceil 0.01 \cdot k + f(k) \rceil \\ h(k) =&x \in {\mathbb {N}} \text { with } \left( {\begin{array}{c}x\\ \lceil \frac{x}{2}\rceil \end{array}}\right) \ge f(k) + g(k) \text { and }\\&\exists ! j \text { with } j < x \text { and } \left( {\begin{array}{c}j\\ \lceil \frac{j}{2}\rceil \end{array}}\right) \ge f(k) + g(k)\\ \end{aligned} \end{aligned}$$

While *f*(*k*) is only an auxiliary function for *g*(*k*), the function *g*(*k*) calculates the number of intermediate blocks to be created using the Gray code. The function *h*(*k*) computes the number of intermediate blocks to be generated by LDPC. The sum $l = k+g(k)+h(k)$ indicates the total quantity of intermediate blocks after the first step. These $k+g(k)$ blocks are then used as inputs to generate the *h*(*k*) LDPC encoded blocks. This ensures that all chunks and previously generated blocks in intermediate steps are captured during LDPC encoding. If the first phase of the implemented Raptor encoding consists of more than two fixed-rate codes, the number of intermediate blocks to be generated must be determined for each of these codes and then, according to the desired order, the chunks together with the preceding intermediate blocks serve as input for the following code.

The $h(k)+g(k)$ intermediate blocks generated in the first step are then encoded into packets using the LT code. The standard procedure is to select the degree of the packets with the predefined distribution function. In contrast to the fountain codes used so far, the final choice of chunks for a packet to use is not distributed equally, but depends on a particular algorithm. Thus, the function may vary depending on the version of the Raptor code.

**Fig. 8:**
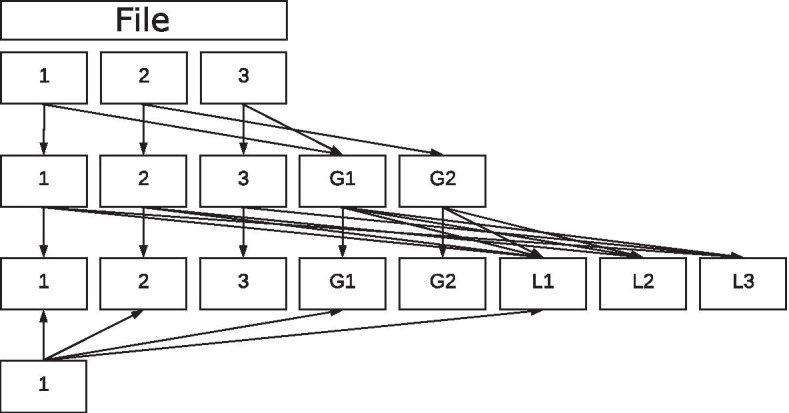
Generation of a Raptor packet with d = 4, a = 5, and b = 1

Figure 8 shows the result of a run of this rateless encoding. The first packet consists of $d=4, a=5, b=1$ of the first chunk ($b = 1$), the fifth chunk ($b = 1 + a\% 11 = 5$), the fourth chunk ($b = ((1 + a)\% 11 + a\% 11) + a\% 11 = 4$) and the second chunk ($b = (((((1 + a)\% 11 + a\% 11) + a\% 11) + a\% 11) + a\% 11) + a\% 11 = 2$).

This procedure limits the size of allowed input chunks (and thus also the final size of the individual packets) to $2^{20} - x$, where *x* is the number of intermediate blocks to be generated in the first step. To avoid this limitation, in RFC 5053 [11] the authors of the Raptor encoding suggest generating ‘sub-blocks’ in addition to chunks (referred to as ‘source blocks’). These are evaluated in the algorithm as a separate run. To enable decoding, only the current chunk number and the number of sub-chunks used must be known. This allows us to encode each chunk separately, offering a selection of almost any number of subdivisions.

The reconstruction of the packets encoded by Raptor codes is quite similar to the Online decoding. The mapping of each intermediate block must be generated exactly in the same way as during encoding. The decoder has to know the number of chunks, the fixed rate codes, and their order. While the number and sequence of most implementation steps is standardized and can therefore be treated as given, a sender must transmit the number of original chunks (or sub-blocks). If the corresponding information is known, the decoding of the Raptor code can also be reduced to a linear equation system and solved using either the Gaussian elimination method [15, 16] or belief propagation [17, 18].

#### Common errors in DNA storage systems

To leverage the rateless property of the described codes, *NOREC4DNA* includes a variety of rules defining error probabilities of DNA sequences.

*Mutations* Depending on the synthesis or storage method, the individual DNA strands are subject to different mutations and mutation probabilities depending on their characteristics. Although all four bases are susceptible to simple mutations, there are differences in the effect, recoverability, and frequency of these mutations. One of the most common mutations is the formation of uracil from cytosine, which, like thymine, would complement adenine and thus would produce a different sequence if sequenced later. A similar defect exists in the oxidation of guanine and the associated formation of 8-oxoguanine. This can bind to both cytosine and adenine and can therefore possibly lead to an error. In addition to a one-by-one mutation, insertions and deletions might lead to further modifications of a DNA sequence.

*Homopolymers* Polymers that contain longer repetitions of the same base are referred to as homopolymers. These fragments are highly unstable during synthesis, polymerase chain reaction (PCR), and subsequent sequencing. On the one hand, due to how next-generation sequencing systems detect the presence of a nucleotide, homopolymers are difficult to correctly sequence [19]. On the other hand, a so-called ‘slippage’ of the enzyme in the region of the homopolymer might occur during PCR (and thus during synthesis and sequencing). Longer homopolymers greatly increase the probability of such an error and thus should be avoided.

*GC Content* The GC content of a DNA strand as shown in Eq. (7) indicates the proportion (i.e., frequency) of the bases guanine and cytosine with respect to the length of the full strand:

7 $$\begin{aligned} \begin{aligned} \text {GC Content} = \dfrac{|G|+|C|}{|G|+|C|+|A|+|T|} \cdot 100\% \text {, }&\text {where |}\star \text {| is the frequency of} \\&\text {base} \star \text {in a given sequence.} \end{aligned} \end{aligned}$$

Since the base pairs A and T always form two hydrogen bonds, whereas the pairs G and C always form three hydrogen bonds, and GC pairs tend to be thermodynamically more stable due to a stack interaction, statistically more frequent errors such as abortions and mutations occur in sequences with low GC content [20, 21]. This effect can be observed in nature, where thermophilic organisms have a significantly higher GC content than comparable species. In nature, depending on the organism, the GC content varies between approximately 20% and almost 80%. To design a stable, synthesizable and sequenceable DNA storage, a balanced GC content of 40–60% is advantageous. This applies to both the overall sequence and the GC content per window.

*Micro-satellites* Also known as ‘short tandem repeats’, two to six base pairs long sequences that are frequently repeated in a DNA sequence are called micro-satellites. Although micro-satellites occur approximately one million times in human DNA, they are still considered unstable due to problems during the sequencing and reproduction process. The most common forms of these error sources are di- and trinucleotide repeats.

Belonging to the class of micro-satellites, dinucleotide repeats consist of a long repetition of two base pairs. The reason for a mutation is the high chance of ‘slippage’ during a PCR process. This causes the newly synthesized strand to form a loop, which is not cut out during the repair attempt but leads to an extension of the strand. Since these locations are not recognized by proof-reading (as correction), they often lead to unrecoverable errors. The more frequently a sequence repeats itself, the higher the probability that during PCR a formation of loops occurs, which then changes the DNA.

Similar to the dinucleotide repeats, this type of error is also based on a repetition of nucleotide sequences. However, as the name implies, repetitions of three nucleotides are considered.

## Implementation

*NOREC4DNA* is written in Python 3 using multiprocessing and a fast C implementation of the most important functions. Additionally, some methods are implemented as CUDA kernels and thus can optionally be executed on GPUs. *NOREC4DNA* is based on an object-oriented approach, i.e., it flexibly supports extensions regarding new methods, coding schemes, metrics, and mutation rules.

### Workflow

**Fig. 9:**
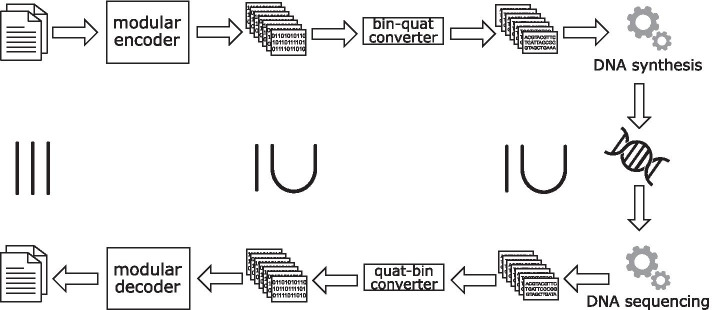
Workflow for storing/retrieving data in/from DNA

The steps to store information in DNA are as follows. First, a number of packets is generated from a chosen (binary) erasure code. A binary-quaternary converter generates a quaternary representation using the bases A, C, G, and T for each packet. The resulting DNA sequences are then synthesized and stored using any suitable method. At a later point in time, these DNA strands are sequenced, translated using the quaternary-binary converter and passed to the decoder as individual packets. The decoder then reconstructs the original file using $(1+\epsilon )*n$ packets. Figure 9 shows the described workflow. It should be noted that the number of DNA strands and thus the number of binary packets received may be smaller than the originally encoded number of DNA strands. We describe the components of the workflow in more detail below.

### Encoder

The encoder divides files into a freely selectable number of chunks (or packets with a selected chunk size) and allows to create an unlimited number of packets. It is possible to either create individual packets or generate a fixed number of packets at once. Furthermore, we added an interface to the decoder implementations. This way, it is possible to generate packets until the selected decoder signals that decoding is possible. The use of a static overhead in combination with a decoder is also possible.

To store additional information about a given file, the encoder allows an optional header chunk to be added during encoding. This artificial chunk can store information such as the file name and the correct length of the padded last chunk.

As an additional feature, a direct enumeration of the contained chunks can be stored per packet. This allows the encoder to operate in an unequal error protection mode [22, 23]. Since the determination of prioritized packets makes a simple reconstruction of the encoded chunks using a seed impossible, an explicit enumeration of the chunk numbers used per packet is required here. This allows a user to specify a list of chunks to decode with high priority while initializing the encoder. These packets can either be encoded into individual packets with degree 1 or have a higher chance to be mixed into packets with a smaller degree (e.g., $\le 4$).

Besides directly using the encoder, *NOREC4DNA* includes multiple scripts to create packets based on full parallel execution. This includes the generation of all possible packets by iterating over all possible seeds in a given seed range. This supports finding the best overall packets for a given input.

Currently, *NOREC4DNA* provides implementations of the following NORECs: LT, Online, and Raptor. The implemented distribution functions are: RobustSoliton, IdealSoliton, as well as the default implementations for Online and Raptor together with a custom ‘adaptable distribution’ that allows a programmatic modification of the distribution function.

### Packet structure

Since the mentioned prioritized packets require an explicit specification of the chunks used, but the LT implementation without prioritization achieves a higher efficiency by specifying the seed, different storage structures are defined for both versions.

**Table 1:** Structure of the different LT packets

(a) *Without explicit specification of chunks*				
#Chunks	Id (Seed)		Data	Checksum
I (4 byte)	I		...	L (4 byte)

(b) *With explicit specification of the chunks*				
#Chunks	Degree	Used chunks	Data	Checksum
I (4 byte)	I	|Packets| $\cdot$ H (2 byte)	...	L (4 byte)

While the packet structure shown in Table 1 may be used as a default setting, the length of each field can be modified. This can be useful if the total number of chunks or the maximum seed fits into a smaller number of bytes (or needs more space). Additionally, static information, such as the total number of chunks the file has been split into, can be omitted during packet creation. While the latter greatly decreases the total and per-packet overhead, this information has to be transmitted out-of-band for a successful decoding. If this information gets lost, the decoding has to test all possible combinations (e.g., all possible number of splits).

The explicit specification of the degree is necessary if the distribution function used and its parameters are not known during decoding. Alternatively, information about the distribution and its configuration could be stored instead.

The function used to generate data for the ‘checksum’ field in Table 1 can be replaced by a (lambda) function. *NOREC4DNA* includes the following pre-defined codes: ‘nocode’, ‘crc8’, ‘crc32’ as well as ‘Reed–Solomon’ with a freely configurable repair symbol size.

While ‘nocode’ works as an identity function and supplies no integrity check for a packet and therefore decreases the overhead, ‘crc8’ and ‘crc32’ introduce overhead, but also provide corresponding integrity checks. Although using a simple checksum might be sufficient to emulate an erasure channel over the DNA medium and thus ensures that only intact packets are decoded, this approach would increase the number of invalid packets even if a single base mutates. Erlich and Zielinski [4] have shown that using a Reed–Solomon code to check integrity and repair errors increases the number of correct packets even if small mutations occur. Since the error probability in a DNA storage system depends on various factors, the number of repair symbols can be chosen freely.

### Bin-quat converter

There are several approaches for encoding binary data using four bases. One variant is the simple conversion and direct storage of the binary data in a quaternary form, i.e., a mapping system as shown in Table 2. The concrete assignment of the bit pairs to the individual bases can be chosen freely.

**Table 2:** Mapping of two bits into quaternary form or DNA base

Bitpair	Quaternary representation	Base
00	0	A
01	1	C
10	2	G
11	3	T

**Table 3:** Mapping: One bit can be encoded into two different DNA bases

Bit	Base
0	A or C
1	G or T

In addition to these obvious approaches, other concepts were studied in the literature. For example, Church et al. [1] stored one bit per base pair, similar to the mapping shown in Table 3. Although this yields a lower information density, it achieves more robustness with regard to mutations and read errors as well as homopolymers. Other methods, such as the approach presented by Limbachiya et al. [24], use only two or three of the four possible bases per block in order to avoid error-prone sequences.

While these mappings can be easily integrated into our framework, *NOREC4DNA* uses the direct mapping approach shown in Table 2. This is due to the ideal information density and the fact that the described fountain codes are used to prevent error-prone sequences. This transformation has been widely used in related work (e.g., [4, 25, 26]).

### Biological rules

To leverage the benefits of NORECs, we introduce a metric ‘error probability’ per packet. This metric represents the probability that a given packet is readable after a full cycle of storing to and reading from DNA. For a flexible and still accurate assessment of these error probabilities, we implemented multiple rule sets. As a reference rule set, the rules as defined by Erlich and Zielinski [4] were implemented. While these rules only focus on homopolymers and the GC content, the remaining implementations add various other rules that can be configured and enabled as required. For example, we offer a fast and reliable C-extension of the most important rules to accelerate the encoding, and a parser-based approach to allow users to easily modify rules without deeper knowledge of Python programming.

Due to the reduction of errors to deletions in the erasure channel, a simulation of mutations over one or more generations (as in [27]) is not necessary. Therefore, it is sufficient to multiply the probability of a mutation of a packet with the number of generations to obtain the probability whether it should be discarded or not.

The following errors were considered and implemented as possible reasons for mutation: homopolymers, unique X-mers, di- and trinucleotide runs, length based errors, A, C, G and T mutation probabilities, (windowed) GC content, illegal symbols, random mutations as well as options for the reverse and reverse complement. Care has been taken to ensure that the errors of synthesis, storage, and sequencing are considered. The used rules for homopolymers, di- and trinucleotides were adapted from the literature [28, 29]. The mutation and error probabilities for the individual bases (e.g., base ‘C’ becomes ‘T’) are mainly chosen based on the work of Grass et al. [29]. Statements about the general random occurrence of errors were compiled from several publications [30, 31].

Equation (8) shows one of the default functions for GC rules. While this distribution for mutation probabilities was roughly adopted from related work [21, 28, 30], these distributions can be replaced during construction of the rule system. This equation returns 0 for all values between 40 and 60 and 1 for all values between 0 and 30 as well as 70 and 100. For the values between 30 and 40 and 60 to 70 this will return a near linear value between 0 and 1.

8 $$\begin{aligned} \begin{aligned} \displaystyle f&= \frac{1}{100} \left( - \frac{x^4}{7200} + \frac{x^3}{36} - \frac{121 x^2}{72} + \frac{175 x}{6} + 100\right) ,\\ \displaystyle err_{gc}&= max(\min (f, 1), 0)\\ \end{aligned} \end{aligned}$$

#### MESA implementation

The highest customizability is obtained by using the MESA [32] API. Since MESA as a web tool for the automated assessment of synthetic DNA fragments and simulation of DNA synthesis, storage, sequencing, and PCR errors does not only allow user-defined configurations but also offers a REST-API, MESA allows a fine-grained and correct assessment of error probabilities per packet. The downside of this approach is the decreased speed introduced through the API.

#### Error prediction accuracy

Since the mutation probabilities of the individual error sources in different scientific studies vary considerably, the implemented default rules have been overestimated in such a way that they create a close upper bound for the actual probability of mutation with the same distribution.

The complexity of finding a universal and accurate error prediction is further increased by the fact that there are various synthesis, amplification, and sequencing methods, all of which are susceptible to different errors and therefore have different mutation and error probabilities. Furthermore, the error probability depends on the duration and the conditions of the information that is stored in DNA. Implemented in this way, long-term storage can be simulated by increasing the probability of errors.

The application of all rules in the default rule set to randomly generated DNA sequences results in an average error probability of about 23% for sequences of length 50 nt (std: 33%, var: 11%, 25-percentile: 0%, 75-percentile: 21%) and about 50% for 164 nt (std: 44%, var: 19%, 25%-percentile: 21%, 75%-percentile: 80%), whereas various articles suggest an error probability of 1-15% (depending on the synthesis, storage, sequencing methods used and most of all, the length of the sequence). However, since we adopted the ratio or distribution of mutations from published data, this overestimation can be adjusted by scaling the error probabilities as required. Additionally, packet-level error correction ensures that small mutations will be repaired and thus will not affect the decoding.

#### Error avoidance during coding

To allow a fast and reliable assessment of the created packets, this metric can be calculated during encoding. All encoders allow the creation of packets based of a strict or weak upper bound. While a strict upper bound simply limits the maximum calculated error probability a generated packet might have, the weak upper bound allows even higher error rates. The weak upper bound might be useful if the given rules are too strict, resulting in all packets to be close to or above the set limit. In detail, this function calculates the error probability and then (equally distributed) draws a number between 0 and 1. If the drawn number is equal or higher than the estimated error probability, the packet is considered valid and will be used, otherwise the packet is discarded. While this approach might yield less optimal packets, this mode will work even when all created packets have high error probability.

### Preprocessing sequenced data

Prior clustering performed in a DNA sequencing pipeline improves the results, since it helps to detect low coverage strands and thus sequences with a higher chance of misreads or mutations. Since *NOREC4DNA* reads FASTA files line by line, sorting the clustered sequences by how often the sequences have been sequenced will allow NOREC4DNA to work more reliably. While most modern sequencing pipelines perform these steps accordingly, we use the Snakemake-based pipeline ‘Natrix’ [33].

### Decoder

Two base classes are provided as implementations of the decoder interface. Apart from the previously mentioned reduction to a Gaussian-solvable linear equation system, we implemented a belief propagation decoder.

#### Gaussian decoder

We implemented a version of Gauss elimination with partial pivoting to achieve a system that is as uniform, fast, and flexible as possible. Since all implemented NORECs work in GF(2)-space and therefore only use the XOR operation, we adjusted and thus accelerated Gaussian elimination. To further increase the decoding speed, we implemented the algorithm in C using Python C-extensions. All developed decoders allow choosing whether decoding should take place after each incoming packet or only after the reception of the last packet using a separate command.

#### Belief propagation decoder

While not particularly needed for a non time-crucial task like the decoding of a file stored in DNA, *NOREC4DNA* includes a belief propagation decoder which might further reduce the decoding time. The algorithm propagates each incoming packet to the already processed packets to both reduce the degree of the new packet and all existing ones. This approach can then transitively propagate changes of reduced packets. The advantage over the Gaussian variant is that the speed of decoding is improved by the temporal distribution of the computations and the possible partial parallelization of this approach.

#### Pseudo decoder

To speed up the use of decoders during encoding, we developed an additional constructor for each decoder, which, taking into account some premises, significantly accelerates this decoding. The idea is to work only on the set of chunks used per packet and ignore the payload of the packets. This is possible because during encoding it is only necessary to determine whether a valid reduction to the identity matrix exists.

For the Gaussian decoder this means that the right side *b* of the equation $Ax = b$ does not have to be solved. It is sufficient to check whether the matrix *A* can be converted into the identity matrix (or the first *n* lines of a $m \times n$ matrix). The belief propagation decoder can reduce the individual packets to Python sets containing the numbers of the chunks used. This makes it possible to map the individual packet reductions using efficient set operators.

## Results

**Fig. 10:**
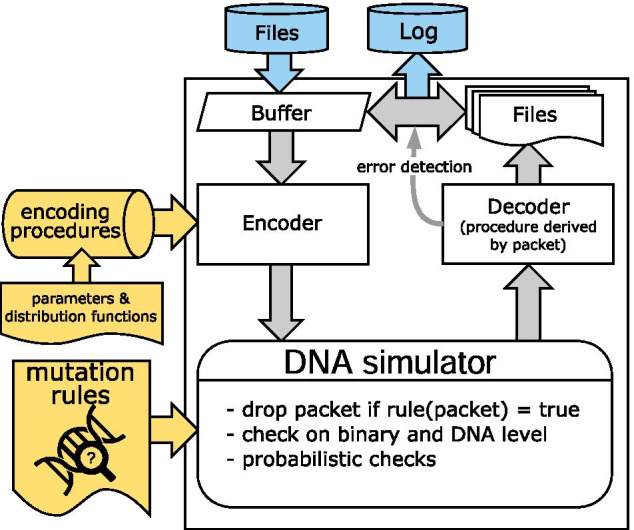
A simulation run

*NOREC4DNA* provides several simulators to compare different codes under various aspects. An illustration of a simulation run is shown in Fig. 10. The freely modifiable and extensible elements that can influence the results of a simulation are highlighted in yellow. The different coding methods have a uniform set of functions in form of a well-defined interface so that they can be exchanged without changing the system.

### Uniformly distributed random error

**Fig. 11:**
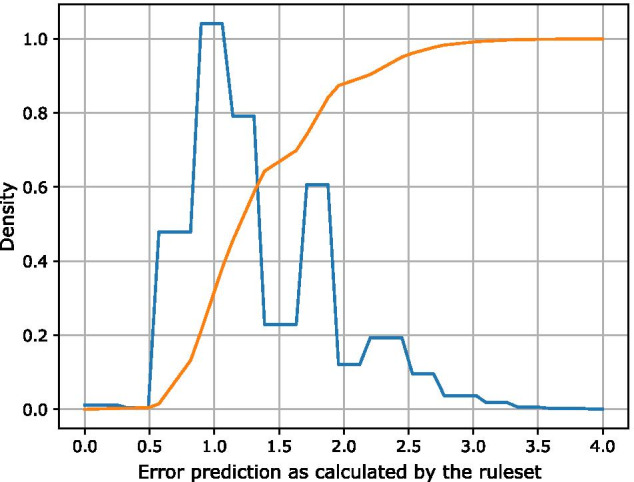
CDF (orange) and PDF (blue) of the error value for 500.000 uniformly random DNA sequences with a length of 120 bp

To show that DNA storage introduces severe restrictions to the possible sequences, we use our default rule set as defined in the class ‘FastDNARules’ to calculate the error prediction of uniformly distributed randomly generated sequences. Figure 11 shows the cumulative density function (CDF) and the probability density function (PDF) of the error prediction for 500.000 uniformly random DNA sequences with a length of 120 bp. The PDF has a peak at an error value of 1.0, i.e., the majority of all sequences have a high error prediction (and thus violate at least one rule). The CDF shows that almost no sequence has an error value of less than 0.5. Thus, most random sequences produce a high error rate for a DNA storage system. This indicates the benefits of NORECs, since they can generate only sequences that yield a low error rate. The rule set can be used to specify user-defined rules and is thus not limited to a maximum of 100%. While each rule on its own may define an exclusion criteria on its own, applying multiple rules on a given sequence will yield an accumulated error value that in some cases will exceed 1.0 (100%). While all sequences with an error value greater than 1.0 violate at least one rule, a higher error prediction indicates that multiple rules were violated for the given sequence. The rule system can (optionally) return an error value per rule to provide more detailed insights.

### Impact of the rules on the packet structure

To test the effects of different encodings (together with additional preselection of packets) on the generated DNA sequences, we encoded an image (the logo of the department as a .jpg file) into packets of the same length of 465 nt using different codes and selected random samples from these created packets.

**Fig. 12:**
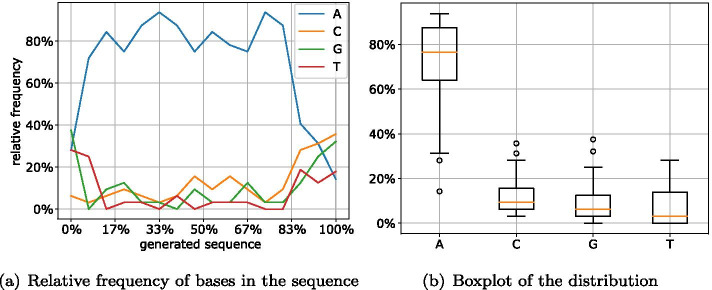
Distribution of bases in a Reed–Solomon packet

In the first run, the packets were created without replacing those with a high error value. In addition, to facilitate a comparison with the implemented NORECs, we analyzed the distribution of the bases for a randomly selected packet of the same length generated by a Reed–Solomon algorithm. Using a sliding window of 32 bases, Fig. 12 shows the relative frequency of the four bases in the generated sequence (0%: start of the sequence; 100%: end of the sequence). Here, the distribution of the individual bases of a Reed–Solomon packet is extremely unfavorable for DNA storage. Compared to the other bases, the relative frequency of base A is well above the majority of the sequence and thus indicates an unfavorable GC content as well as a high chance of homopolymers. This is due to the fact that Reed–Solomon is a systematic code, which in our case means that the encoded file has an unfavorable distribution regarding the selected rules. It has to be mentioned that the image as the chosen input file has longer sequences of the same symbols, which results in a corresponding distribution in the generated DNA strand.

This also shows that an implementation of the Raptor code in the form of a systematic code would not have a positive impact on the created packets. In previous DNA storage approaches (e.g., [2, 31]), this problem is combatted by intermediary codes with appropriate substitutions (using Huffman coding, scrambling, or others). However, these mitigations require more computing effort and usually reduce the storage density by inserting additional symbols or lack generalization.

**Fig. 13:**
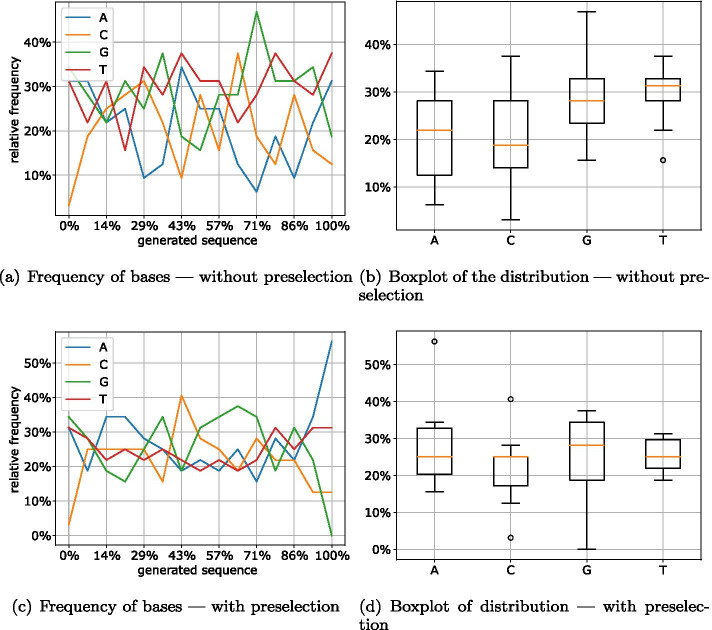
Distribution of bases in LT packets (Robust Soliton)

Figure 13 shows the distribution of the individual bases in a randomly selected LT packet with Robust Soliton distribution. While the distribution of packets without preselection (13a, b) shows strong fluctuations for the individual bases, the randomly selected packet generation with preselection (13c, d) shows significant improvements. The distribution of the bases shows that the selected packet has a nearly optimal distribution of 25% per base (Fig. 13d). Only for the start and end of the DNA sequence there are major differences in the frequency of the individual bases. However, this can be attributed to the random seed and the error detection/correction attached at the beginning and the end of the sequence. The desired 25% are motivated by the ideal distribution of the nucleotides in each window. By having an equal distribution of 25%, the sequence is less likely to contain homopolymers and will also have a balanced GC content of around 50%.

**Fig. 14:**
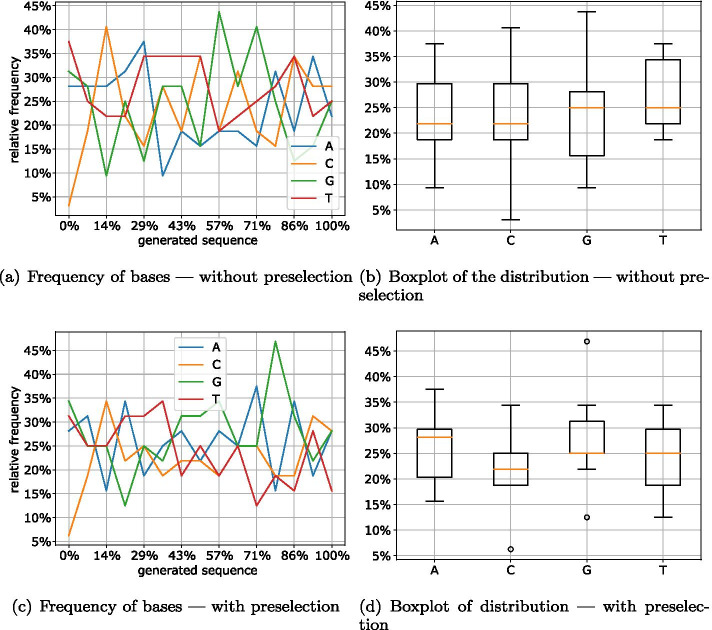
Distribution of bases in online packets (quality = 5)

For the Online encoding, this analysis delivers similar results. The $\delta$ parameter was chosen such that the packets have the same size as the LT and Raptor encoding. While the improvement between the two variants (Fig. 14a, c) is not apparent at first glance, the boxplots (Fig. 14b, d) of the distribution of the four bases show that the version with preselection has a much smaller variance.

**Fig. 15:**
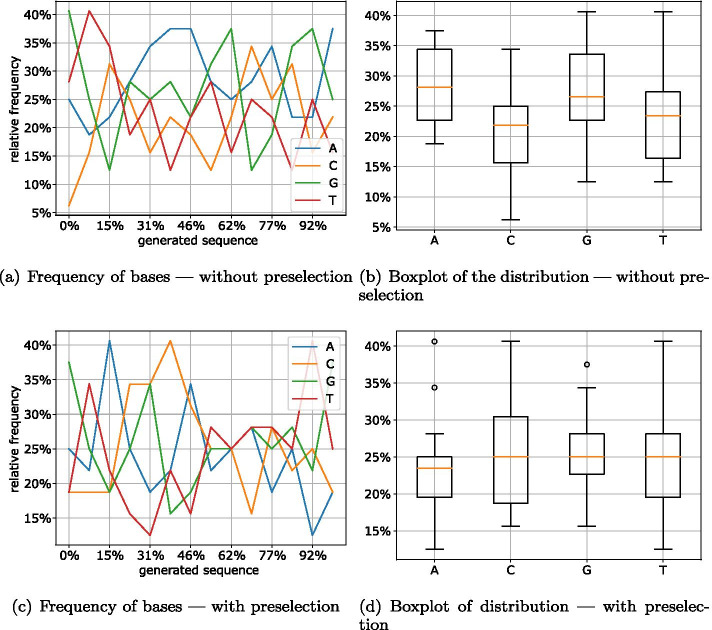
Distribution of bases in Raptor packets

For the Raptor encoding shown in Fig. 15, preselection apparently has the slightest impact. This is due to the fact that Raptor already achieves good results with the non-optimized packets. Thus, apart from the beginning of the DNA sequence, the occurrence of all bases is between 13% and 36%. However, except for base A, after preselection all bases average at the optimum of 25% (Fig. 15d).

The very low C content, which can be clearly recognized at the beginning of all examined packets, can be explained by the fact that the header is stored here. Since the header contains the parameters ‘Number of Chunks’ and ‘Seed’, and the number of chunks is the same for all packets, the same value is stored at this location. To mitigate the formation of homopolymers for the number of chunks field, we can apply a mask or reduce the size of the field. Another solution could be to make a different choice for the number of chunks or not to write them into the header but to transmit them out of band. Using a more work-intensive decoding, this information could even be omitted completely. Alternatively, this field could be encoded using regular codes for DNA storage. This would reduce the information density by a small percentage but would guarantee that all inputs satisfy the DNA storage requirements.

We want to emphasize that the selected packets in our evaluations, without loss of generality, were drawn at random. Graphs for other packets of this experiment can be found in the Github repository or generated using the scripts provided.

### Estimated error probabilities of the codes

To further analyze the ability to create sequences with low error probabilities, we created all possible sequences for a given file with a fixed sequence size. As an input we used a text file containing the German fairytale ‘Sleeping Beauty’ to create 163 nt long sequences. With a 2 byte seed (and a 2 byte Reed–Solomon code at the end of each sequence), we created 65,536 sequences using each code and analyzed these sequences with the default rule set.

**Fig. 16:**
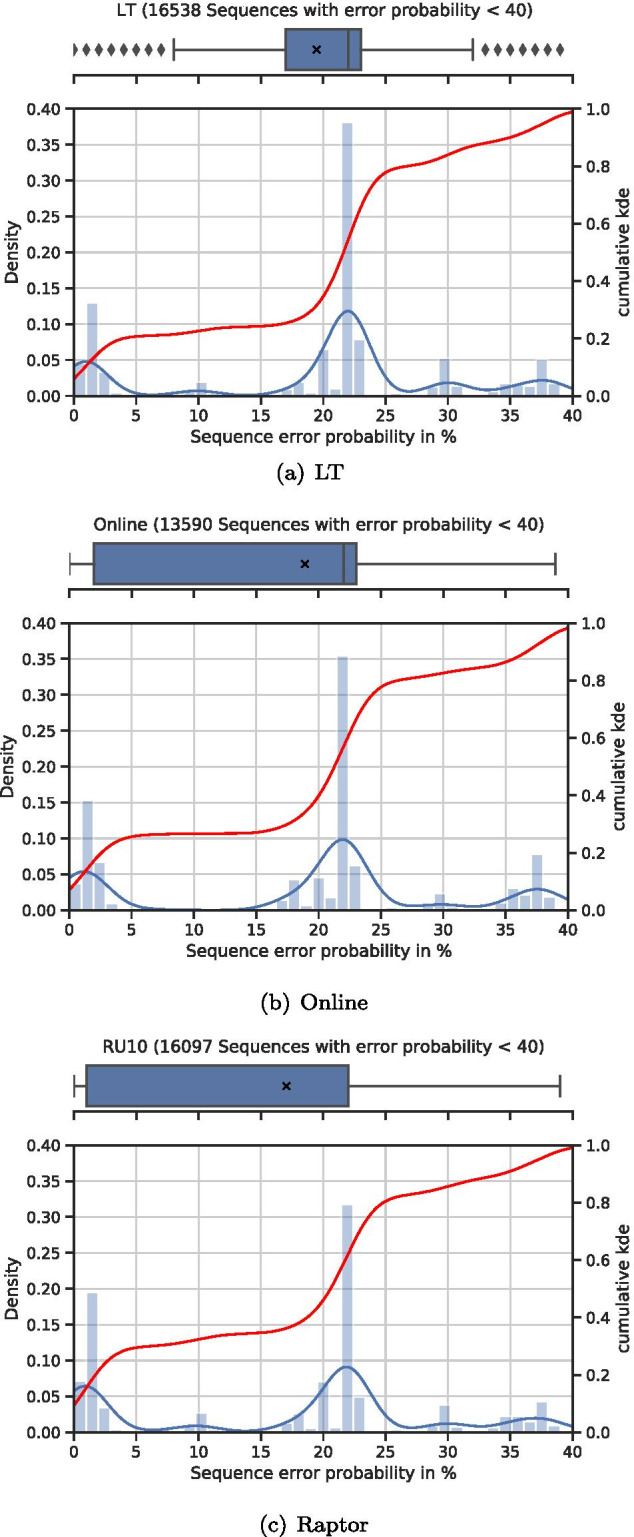
Distribution of the error probability of created sequences

Figure 16a shows the distribution of the error probabilities of all 16,538 sequences that have an error probability of less than 40%. With a mean value (black ‘X’) of nearly 20% error probability and a tight lower and upper quartile of 17% and 23%, respectively, we see that LT performs worst. For LT, the cumulative kernel density estimate (red) shows that not even 33% of the packets have an error probability of less than 20%. Figure 16b shows that the Online code generates only 13,590 sequences in the specified range, but does not only have a slightly lower mean error probability (and lower quartile) of 19% (and 2%) but also yields nearly 40% sequences with an error probability below 20%.

The distribution of the sequences generated using the Raptor code, as shown in Fig. 16c, shows a further improvement. While the basic distribution is similar to the sequences created using the Online code, there are major differences: (a) the number of sequences with an error probability of less than 40% is 16,097 and thus higher than for the Online code, and (b) there are more sequences with a low error probability (and especially 0%). In more detail, the mean value is 18%, and the lower and upper quartiles are 1% and 22%, respectively. By looking at the cumulative kernel density estimate, we can see that 45% of the sequences in this range have an error probability below 20%.

While the given file was split into 163 (196 for Online, due to the larger header) chunks and thus $(1+\epsilon )*163$ ($(1+\epsilon )*196$ for Online) sequences would be sufficient to decode the reconstructed the file, a large number of sequences with a low error probability is crucial for larger files with an equal or smaller sequence length. Since the seed is usually stored in a 2 byte field, we are limited to 65,535 different packets. If the code generally produces many low error sequences, we can use the 2 byte field for larger files than for a code that produces more high error sequences. While we could and might have to increase the size of the seed field to 4 (or even 8) bytes, this will introduce an overhead and thus will decrease the storage density (and will increase the cost). Since LT and Online (to some extent) are more susceptible to the coupon collector’s problem, these codes will generally require a larger $\epsilon$ than Raptor.

### Susceptibility to the coupon collector’s problem

The coupon collector’s problem indicates the complexity of finding all pieces required to own a complete set of pieces (i.e., decoded source symbols). Using it, we can analyze how well the codes perform regarding the (average) overhead required to successfully decode the original data. For the LT code, Luby states that an average of $k + O(\sqrt{k}\ln ^2(k/\delta ))$ encoded symbols are required to decode a file that was split into *k* chunks with a probability of $1-\delta$ [6]. Online [7] as well as Raptor [8] achieve a theoretical linear complexity and thus require a flat $(1+\epsilon )*k$ (with $\epsilon \rightarrow 0$ when $k \rightarrow \infty$) sequences to reconstruct the input file. This is due to the precoding applied in both codes.

**Fig. 17:**
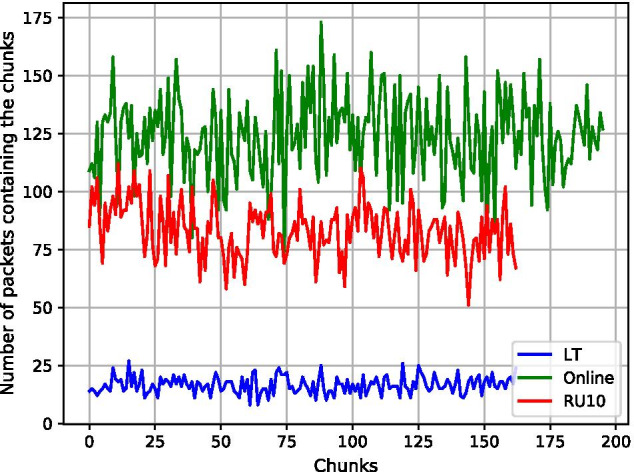
Number of packets containing each chunk (based on 500 packets each)

Figure 17 shows the number of times each chunk of the input files is present in a created packet. Although we analyzed 500 packets for an encoding with only 196 chunks, for LT almost no chunk is included into more than 25 packets. This means that there is (a) a higher chance of a chunk missing due to errors and mutations (a chunk might have a high error rate and propagate this error probability to created packets including this chunk), and (b) a higher susceptibility to the coupon collector’s problem. Since each packet includes only a few chunks, the probability of a chunk not being included into any packet already read increases. In contrast, Online and Raptor with a minimum of 50 and a maximum of over 110 occurrences per chunk (75 and 175 for Online) are less likely to suffer from these problems. It should be noted that due to the larger header used for Online and the decision to use the same chunk size instead of the same number of chunks (for the comparisons described earlier), the input file for the Online code was split into 196 chunks and thus cannot be compared directly. The Online code calculates the number of auxiliary blocks based on the number of chunks and thus has a higher average number of packets containing each chunk than Raptor.

Figure 17 illustrates why the Online and Raptor codes yield better results regarding the impact of the rules on the packet structure, as already described in “Impact of the rules on the packet structure” section. Since both codes combine more chunks to create packets, they are more likely to combine different symbols using XOR and thus create an equal distribution of all nucleotides. Logically, this effect only occurs if the input file is not perfectly random (otherwise, the LT code would also yield packets with a nearly perfect 25% distribution for each base).

### Encoding speed

In this section, we evaluate the encoding speed of *NOREC4DNA*. To reduce the encoding speed, we applied various techniques including multi-threading and Python’s C-extensions. All following experiments were performed using a server with 2x Intel(R) Xeon(R) CPU E5-2698 v4 @ 2.20GHz (40 Cores, 80 Threads) and a total of 252 GB RAM.

Since the entire process of storing data in DNA is still expensive and error-prone, it is well advised to generate all possible sequences for a given input file using the whole seed range. By sorting these sequences according to the estimated error values, we can guarantee that we synthesize the best possible sequences.

In a first experiment, we used the “find_minium_packets” script with the RU10 encoder to create packets. Each file was split into chunks of 34 bytes each, and a header chunk was added. Additionally, for all packets, a Reed–Solomon (RS) code of 4 (6) bytes was added to detect and repair inner packet errors. This resulted in sequences of length 160 nt (168 nt for the experiments with a 6 bytes Reed–Solomon code). After we created and tested all packets, we merged the results and sorted them according to their error values. For our error prediction, the default rules defined in the framework were used. Each experiment was repeated 50 times.

**Fig. 18:**
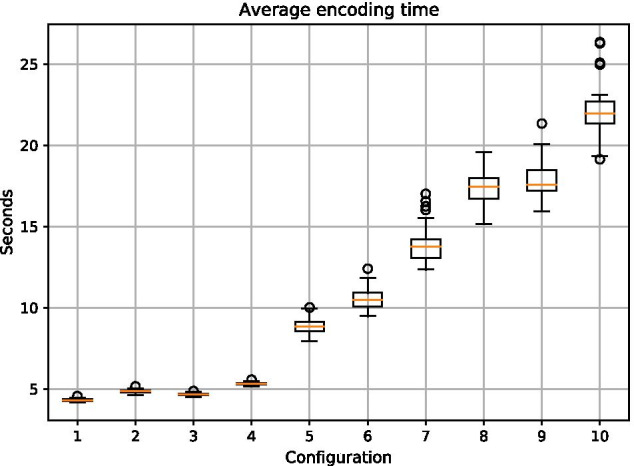
Time required to create 65,536 packets and sorting them according to the error predictions for different configurations

**Table 4:** Configurations as shown in Fig. 18

Config 1	Config 2	Config 3	Config 4
1 kb Lorem—4 RS	1 kb Lorem—6 RS	4.9 kb fairytale—4 RS	4.9 kb fairytale—6 RS

Config 5	Config 6	Config 7	Config 8
34 kb LICENSE—4 RS	34 kb LICENSE—6 RS	72kb logo—4 RS	72 kb logo—6 RS

Config 9		Config 10	
100 kb Lorem—4 RS		100 kb Lorem—6 RS	

All files can be accessed in the source repository

As shown in Fig. 18 (and Table 4), the time needed for small files (Configurations 1 - 4) does not increase significantly. Both the 1 kb “Lorem Ipsum” text file and the 4.9 kb “Sleeping Beauty” fairytale file take nearly the same time to encode and only differ when the number of Reed–Solomon symbols increases. This is due to the increased size of the sequences and thus more effort for error calculation.

For the AGPL-LICENSE file (Configurations 5–6; 34 kb) and the image “logo.jpg” (Configurations 7–8; 72 kb), we obtained a higher encoding time for an increased file size and a higher number of Reed–Solomon symbols. For the 100 kb version of “Lorem Ipsum” (Configurations 9–10, 100 kb), there is nearly no difference between the 6 bytes Reed–Solomon version of the “logo.jpg” and the 4 bytes Reed–Solomon version (Configuration 8 vs. Configuration 9). However, the average encoding time for the version with 6 bytes of Reed–Solomon per packet (Configuration 10) increases to about 22 seconds. By comparing the 1 kb Lorem Ipsum file with the 100 kb version (Configuration 1 and Configuration 9), we see that with an increase from an average of 4.5 s for the 1 kb file and 17.5 seconds for the 100 kb file, the time required for encoding does not significantly increase with the input file size. We additionally conducted this experiment with a 1 Mb file containing random ASCII characters using 4 Reed–Solomon symbols per packet. It took an average of 127 s to finish.

To investigate the encoding speed further, we profiled the multi-threaded encoding process. This showed that across all files tested, approximately 76% of the computation time is used for sorting and merging the created packets. Thus, optimizations of sorting and merging could greatly reduce the encoding time. To reduce the speed impact of sorting and merging, a user should define the required packets and the maximum tolerated error before calculating all possible packets.

A further possibility to improve the encoding speed is to use cloud services to take advantage of the highly parallel nature of the encoding scheme (given the same input, the encoding could easily be performed using multiple computers in a parallel or distributed manner.)

Thus, there are several options for improving the encoding speed. However, taking the price and required time for synthesis and sequencing into account, the current speed is already sufficient for real-world usage. Even for larger files, *NOREC4DNA* can generate sequences in a timely fashion in the order of seconds or minutes.

## Conclusion

We presented *NOREC4DNA*, a software framework to use, test, compare, and improve NORECs for DNA storage systems. We showed that such fountain coding schemes can effectively be used to satisfy the restrictions associated with the DNA medium. Additionally, these codes can adapt to the possible variable lengths of DNA strands and have nearly zero overhead. Therefore, using NORECs in DNA storage systems helps to achieve the goal of building a robust and high capacity long term storage.

Although our experimental comparisons only approximate storing data in synthesized DNA, the obtained results are very promising. Our evaluations showed that especially Raptor codes that have not yet been used for DNA storage systems yield excellent results. In addition, Online codes are quite useful for DNA storage systems. Both codes show better results than LT codes that were already used for storing data in DNA by Erlich and Zielinski [4]. Since these authors have obtained good results with LT codes for DNA storage, it is likely that Raptor and Online codes will achieve significant improvements over LT codes for such storage systems.

There are several areas for future work. For example, we plan to improve the distribution functions for the used NORECs to tailor them to the DNA specific error channel. Furthermore, we will investigate how enforced rules can be used to improve the error correcting abilities of codes during DNA sequencing. Finally, we intend to create novel NORECs that are optimized for the special restrictions and limitations of DNA storage.

## Availability and requirements

- Project name: NOREC4DNA
- Project home page: https://github.com/umr-ds/NOREC4DNA
- Operating system(s): Platform independent
- Programming language: Python 3.6 or above, C
- Other requirements: Python3, Python3-dev or Docker
- License: AGPL v3.0
- Any restrictions to use by non-academics: N/A

## Data Availability

The code is released with an open source license and can be accessed at: https://github.com/umr-ds/NOREC4DNA

## Abbreviations

DNA: Deoxyribonucleic acid
NOREC: Near-optimal rateless erasure code
LT: Luby transform
LDPC: Low density parity check
PCR: Polymerase chain reaction
RS: Reed–Solomon
CDF: Cumulative density function
PDF: Probability density function
